# Dysfunction in superior frontal gyrus associated with diphasic dyskinesia in Parkinson’s disease

**DOI:** 10.1038/s41531-020-00133-y

**Published:** 2020-10-30

**Authors:** Yu-Ting Shen, Yong-Sheng Yuan, Min Wang, Yan Zhi, Jian-Wei Wang, Li-Na Wang, Ke-Wei Ma, Qian-Qian Si, Ke-Zhong Zhang

**Affiliations:** 1 Department of Neurology, The First Affiliated Hospital of Nanjing Medical University, Nanjing, China; 2 Department of Neurology, Changshu No.1 People’s Hospital, Suzhou, China; 3 Department of Radiology, The First Affiliated Hospital of Nanjing Medical University, Nanjing, China

**Keywords:** Parkinson's disease, Parkinson's disease

## Abstract

Alterations in brain function in Parkinson’s disease (PD) patients with diphasic dyskinesia have not been investigated. We aimed to explore the alterations in regional brain function. Each of 53 levodopa (LD)-treated PD patients had two resting-state functional magnetic resonance imaging (rs-fMRI) scans in the same morning, before and after taking LD. The regional homogeneity (ReHo) approach was used to reveal local synchronization changes. Two-way factorial repeated measures analysis of covariance, with group as a between-subject factor and LD effect as a within-subject factor, was performed to explore the two main effects and interaction. Interactive analysis was used to show outcomes that combined disease status and LD effect. Spearman’s correlations were used to detect associations between interactive brain regions and severity of dyskinetic symptoms, assessed by the Unified Dyskinesia Rating Scale (UDyRS) scores, along with moderation analyses. There was no significant difference in the main group effect analysis. Significantly different clusters obtained from main LD effect analysis were in left caudate nucleus and putamen. ReHo values decreased in the caudate nucleus and increased in the putamen during the ON state after taking LD. Interaction between group and LD effect was found in left medial superior frontal gyrus (mSFG), where there were the lowest ReHo values, and was negatively correlated with UDyRS scores in the diphasic dyskinetic group during the ON state. The relationship was independent of LD dose. Abnormal local synchronization in the mSFG is closely associated with the development of diphasic dyskinesia in PD patients.

## Introduction

In levodopa-induced dyskinesia (LID), which is a common motor complication in Parkinson’s disease (PD), levodopa (LD) has been confirmed to be the major contributor to abnormal involuntary movements^1^. LID can be subdivided into peak-dose dyskinesia, diphasic dyskinesia, and off-period dystonia^1,2^. Peak-dose dyskinesia is the most common motor complication, followed by diphasic dyskinesia^1^. Dyskinetic symptoms in patients with peak-dose dyskinesia appear at the peak of the LD effect, whereas diphasic dyskinesia arises at the beginning or end of the effect^1,2^. Diphasic dyskinesia tends to be associated with aberrant function in more brain regions, and is reported to be more troublesome than peak-dose dyskinesia^1^. Several studies have explored the functional changes in relevant brain regions in peak-dose dyskinetic patients^3–7^, but to the best of our knowledge, alterations in brain function in patients with diphasic dyskinesia have not been investigated. An understanding of alterations in brain function in diphasic dyskinesia may help search for effective new treatments.

In the current study, we used regional homogeneity (ReHo) to explore regional brain pathology in PD patients with diphasic dyskinesia. ReHo is a data-driven method, which can reveal changes in temporal neural activities in different brain regions^8^. We hypothesized that aberrant regional synchronization in some brain areas, especially those associated with somatic motor function, might play a role in abnormal involuntary movements in PD patients with diphasic dyskinesia.

## Results

### Demographic and clinical data

Demographic characteristics and clinical evaluations of PD patients with diphasic dyskinesia and without dyskinesia are provided in Table 1. No significant effect was observed for age, gender, or education years. There was also no significant difference in disease duration, UPDRS-III score, H&Y stage, LD equivalent daily dose (LEDD) or Mini Mental State Examination (MMSE) between PD patients with diphasic dyskinesia and those without dyskinesia. In summary, both demographic characteristics and corresponding clinical assessments were well matched between the two subgroups of PD patients.

**Table 1:** Demographic and clinical data for PD patients with diphasic dyskinesia and without dyskinesia.

Items	PD patients with diphasic dyskinesia	PD patients without dyskinesia	*P* values
	(*n* = 30)	(*n* = 23)	
Age (years)	63.83 ± 9.08	61.52 ± 8.23	0.34^a^
Gender (F/M)	13/17	8/15	0.53^b^
Education (years)	10.23 ± 3.88	10.57 ± 2.69	0.87^c^
Disease duration (years)	9.80 ± 4.63	9.09 ± 3.32	0.50^c^
UPDRS-III (OFF state)	36.80 ± 13.51	34.30 ± 17.53	0.56^a^
H&Y stages	2.47 ± 0.59	2.27 ± 0.81	0.39^c^
LEDD	743.67 ± 290.21	706.90 ± 399.64	0.43^c^
MMSE	27.70 ± 1.97	27.96 ± 2.01	0.54^c^
UDyRS	52.43 ± 30.10	NA	NA

*PD* Parkinson’s disease, *F* female, *M* male, *UPDRS-III* motor component of Unified Parkinson’s Disease Rating Scale, *H&Y* Hoehn and Yahr stages, *LEDD* levodopa equivalent daily dose, *MMSE* mini mental state exam, *UDyRS* Unified Dyskinesia Rating Scale, *NA* not applicable.

Values were expressed as mean±standard deviation.

^a^Two-sample *t* test.

^b^Chi-square test.

^c^Mann–Whitney *U* test.

### ReHo analysis

**Fig. 1:**
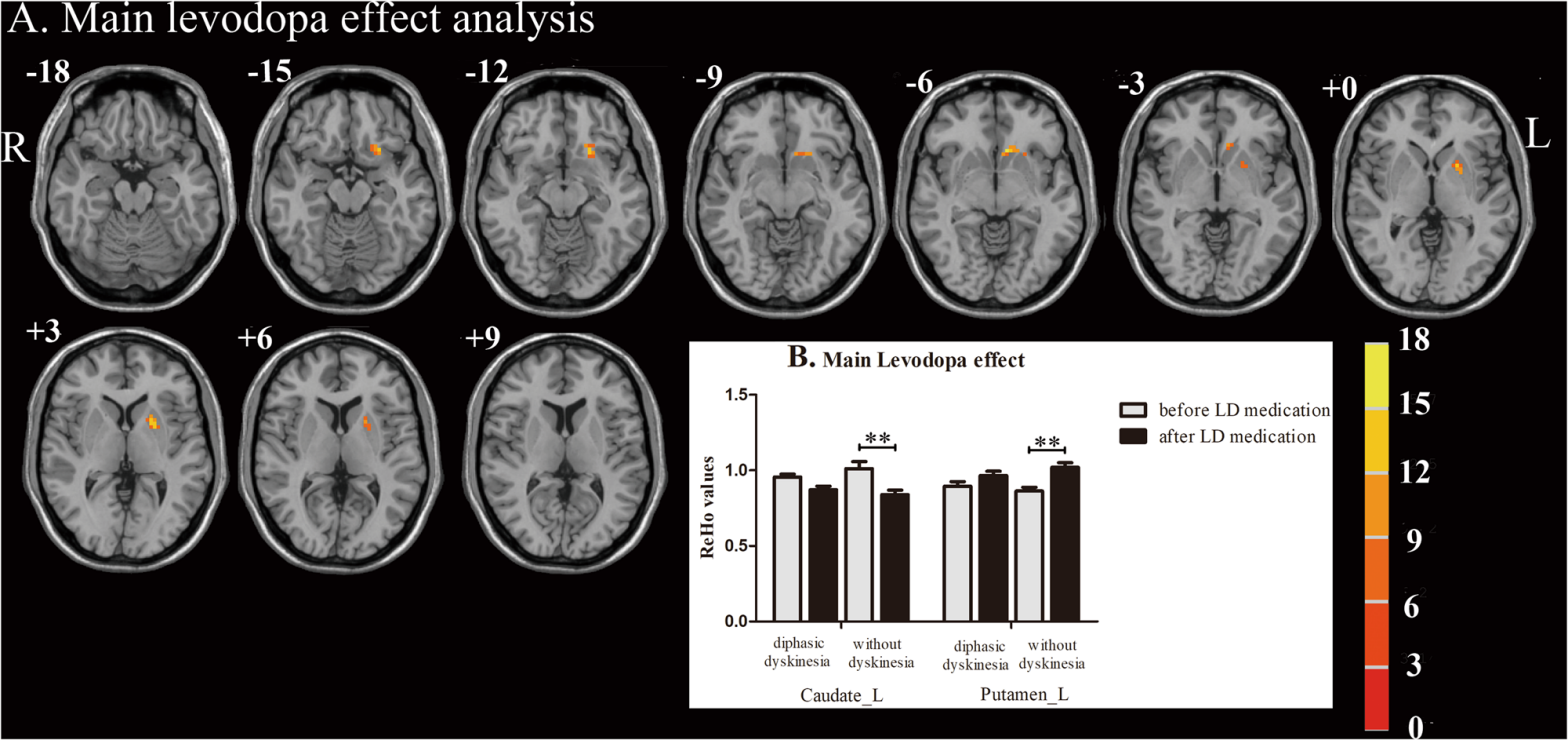
Main effect of levodopa. **a** Significantly different clusters obtained from the main effect of levodopa (before or after taking levodopa medication) were in the left caudate nucleus and putamen. The color bar indicates F values from repeated measures analysis of covariance (ANCOVA), with group (PD patients with diphasic dyskinesia or PD patients without dyskinesia) as a between-subject factor and levodopa effect (before or after taking levodopa medication) as a within-subject factor, adjusting for age, gender, and education years. Thresholds were set at a corrected *p* < 0.01, determined by Monte Carlo simulation. **b** **Post hoc tests were corrected by Bonferroni correction with a significant different *p* < 0.013 (0.05/4 [number of pair‐comparisons]). Error bars indicate standard deviations. PD Parkinson’s disease, ReHo Regional homogeneity, R right, L left.

**Fig. 2:**
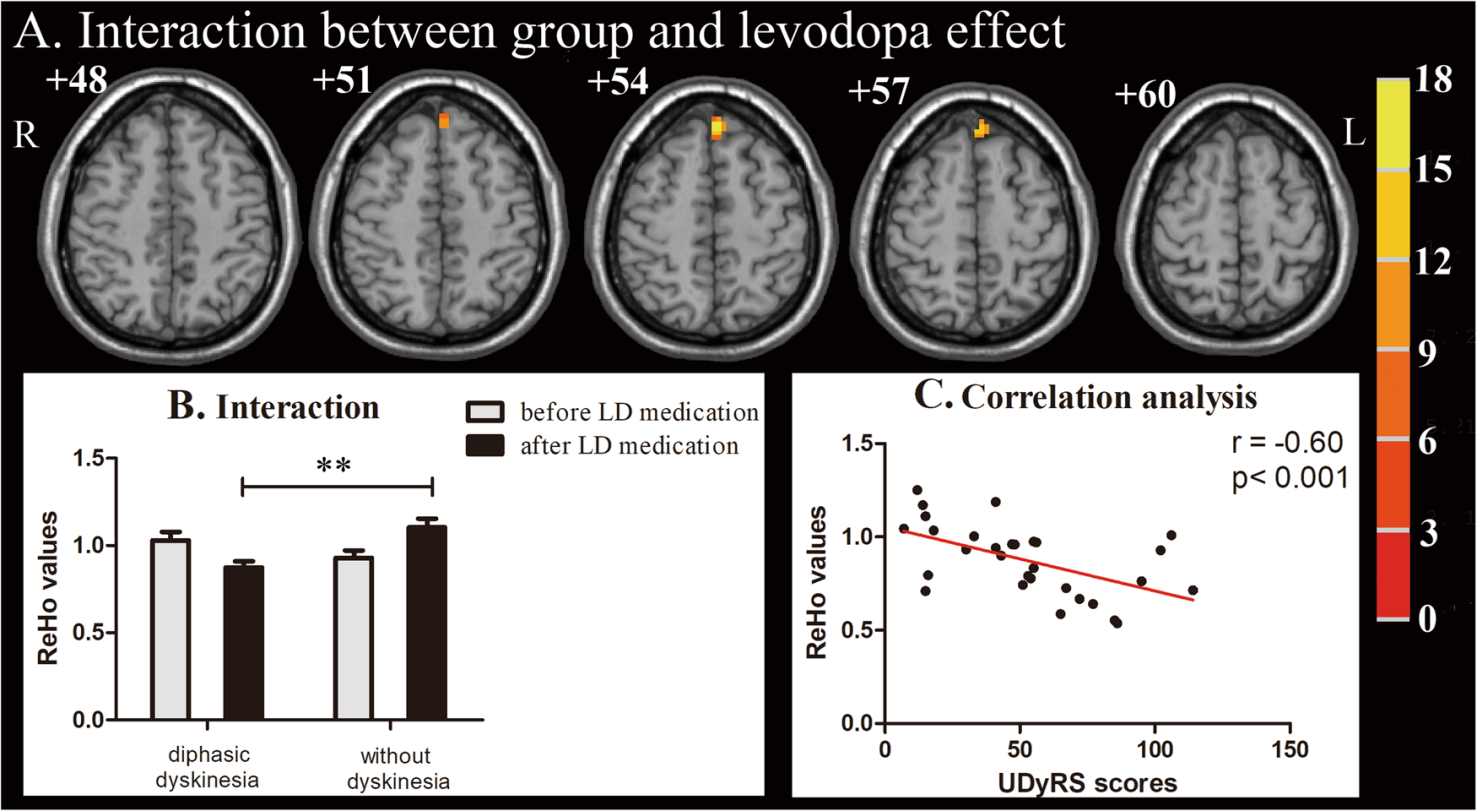
Interaction between group and levodopa effect, along with correlation analysis. **a** Interaction between group and levodopa effect was found in the left medial superior frontal gyrus (mSFG). Thresholds were set at a corrected *p* < 0.01, determined by Monte Carlo simulation. The color bar indicates *F* values from repeated measures analysis of covariance (ANCOVA), with group (PD patients with diphasic dyskinesia or PD patients without dyskinesia) as a between-subject factor and levodopa effect (before or after taking levodopa medication) as a within-subject factor, adjusting for age, gender, and education years. **b** **Post hoc tests were corrected by Bonferroni correction with a significant different *p* < 0.013 (0.05/4 [number of pair‐comparisons]). Error bars indicate standard deviations. **c** ReHo values in left mSFG were negatively associated with UDyRS scores in diphasic dyskinetic patients. PD Parkinson’s disease, ReHo regional homogeneity, R right, L left; UDyRS Unified Dyskinesia Rating Scale.

The effects of dyskinesia and LD effect on ReHo values in PD patients with diphasic dyskinesia, and those without dyskinesia, are shown in Table 2 and Figs 1 and 2. There was no significant difference in the main group effect (with group as a between-subject factor). In other words, irrespective of medication, ReHo in the whole brain of PD patients with diphasic dyskinesia was the same as that in PD patients without dyskinesia. Significantly different clusters obtained from the main LD effect (with LD effect as a within-subject factor) were in the left caudate nucleus (*F* = 17.22, *p* < 0.01, corrected) and putamen (*F* = 15.57, *p* < 0.01, corrected) (Fig. 1A). This means that after taking antiparkinsonian drugs, there was the same trend towards change of ReHo values in the left caudate nucleus in patients with diphasic dyskinesia (*p* = 0.061, corrected) as in patients without dyskinesia (*p* = 0.003, corrected). Alterations in ReHo values in the left putamen of patients with diphasic dyskinesia (*p* = 0.074, corrected) were the same as those in patients without dyskinesia (*p* = 0.001, corrected). Specifically, ReHo values in the left putamen increased, whereas ReHo values in the left caudate nucleus decreased (Fig. 1B). Interaction between group and LD effect was also found in the left medial superior frontal gyrus (mSFG) (*F* = 15.64, *p* < 0.01, corrected) (Fig. 2A). Videlicet, after taking antiparkinsonian drugs, the trend towards altered ReHo values in the left mSFG of PD patients with diphasic dyskinesia was notably different from that in patients without dyskinesia. In PD patients with diphasic dyskinesia, ReHo values in the left mSFG showed a trend towards decrease after taking antiparkinsonian drugs (*p* = 0.057, corrected) whereas, in patients without dyskinesia, ReHo values in the left mSFG showed a trend towards increase after taking antiparkinsonian drugs (*p* = 0.064, corrected). After taking antiparkinsonian drugs, ReHo values in the left mSFG were significantly lower in PD patients with diphasic dyskinesia than in patients without dyskinesia (*p* = 0.003, corrected) (Fig. 2B).

**Table 2:** Group × levodopa effect ANCOVA of ReHo.

Brain region (AAL)	Peak MNI			Peak	Cluster size
	Coordinates x, y, z (mm)			F value	(voxels)
(1) Main effect of group					
None					
(2) Main effect of levodopa					
Caudate_L	−9	21	−6	17.22	32
Putamen_L	−21	9	3	15.57	24
(3) Groups × levodopa effect interaction					
Frontal_Sup_Medial_L	−3	39	54	15.64	26

Repeated measures analysis of covariance (ANCOVA), with group (PD patients with diphasic dyskinesia or PD patients without dyskinesia) as a between-subject factor and levodopa effect (before or after taking levodopa medication) as a within-subject factor, was performed after adjusting for age, gender, and education years, to explore the two main effects and interaction. All statistical thresholds were set at a corrected *p* < 0.01, determined by Monte Carlo simulation for multiple comparisons.

*PD* Parkinson’s disease, *ReHo* regional homogeneity, *AAL* anatomical automatic labeling, *MNI* Montreal Neurological Institute, *L* left, *Sup* superior.

### Correlation analysis

Correlation analysis of interactions between group and LD effect indicated that ReHo values in the left mSFG were negatively associated with UDyRS scores (*r* = −0.60, *p* < 0.001) in diphasic dyskinetic patients (Fig. 2C).

### Moderation analysis

**Fig. 3:**
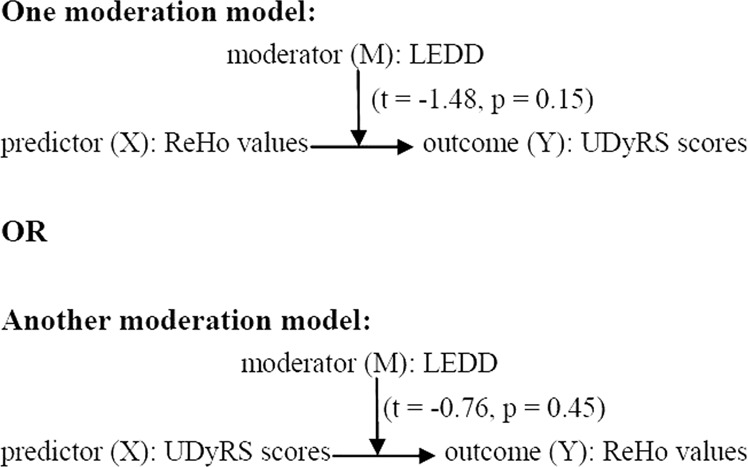
Moderation analysis. LEDD did not significantly moderate the association between ReHo values in the left medial superior frontal gyrus (mSFG) and UDyRS scores, regardless of whether UDyRS scores were taken as predictor (*X*) (*t* = −0.76, *p* = 0.45) or outcome (*Y*) (*t* = −1.48, *p* = 0.15). LEDD Levodopa equivalent daily dose; ReHo Regional homogeneity, UDyRS Unified Dyskinesia Rating Scale.

Moderation analyses showed that LEDD did not significantly moderate the association between ReHo values in the left mSFG and UDyRS scores, regardless of whether UDyRS scores were taken as predictor (*X*) (*t* = −0.76, *p* = 0.45) or outcome (*Y*) (*t* = −1.48, *p* = 0.15), as shown in Fig. 3. In other words, the relationship between ReHo values and UDyRS scores was independent of LD dose.

## Discussion

Our study provides a new perspective on the neural mechanisms underlying diphasic dyskinesia using ReHo, an approach that can be used to examine the complex effects of local functional specialization^9^.

Analysis of the main LD effect showed that ReHo values increased in the left putamen and decreased in the left caudate nucleus after taking antiparkinsonian drugs, including LD. LD, the precursor to dopamine, is the most effective medication to improve motor symptoms in PD patients^10^. Dopamine has a role in modulating firing of medium spiny neurons in the striatum, including the putamen and caudate nucleus^11,12^. Dopamine has opposite effects on the direct and indirect pathways^13^, and between cortical areas and the striatum pathway, and ultimately enhances movement^14^. The putamen, which is especially seriously affected by dopaminergic denervation in PD^15^, has been shown to be activated after taking LD^16^. This is consistent with our own finding that ReHo values in the left putamen increased when LD was effective. Conversely, ReHo values in the left caudate nucleus decreased after taking antiparkinsonian drugs. It has been proposed that the caudate nucleus may be involved in response inhibition^17,18^. Impaired suppression in subcortical regions may contribute to hyperactivation in cortical regions^19^. Previous studies have also shown that appropriate corticostriatal connectivity may be restored after dopaminergic replacement therapy^20,21^, which helps to normalize disrupted brain networks^22^. Notably, ReHo values in either the putamen or caudate nucleus were significantly changed in patients without dyskinesia but only showed a trend towards change in patients with diphasic dyskinesia. This may be because of lack of regularity in the appearance of diphasic dyskinetic symptoms following LD medication. In our study, patients with diphasic dyskinesia received the ON-state fMRI scan just as dyskinetic symptoms were expected to occur, based on previous diaries, which means that this occurrence of dyskinetic symptoms may not be typical.

Most importantly, the interactive analysis revealed that ReHo values in the left mSFG of patients with diphasic dyskinesia decreased after taking antiparkinsonian drugs, and were negatively correlated with UDyRS scores. UDyRS is a comprehensive evaluation scale for dyskinesias in PD and reflects the severity of dyskinetic symptoms with acceptable internal consistency, inter- and intra-rater reliability and temporal stability^23,24^. Less synchronization in the mSFG was thus associated with more serious dyskinetic symptoms. Converging lines of evidence have also confirmed that SFG has an important role in regulation of response inhibition^25–27^. Deficient inhibitory control or dysfunctional inhibitory processes may contribute to overly impulsive behavior^28^, with similar pathological mechanisms to LID^6,29^. Specifically, the SFG has been shown to have a specific role in the control of impulsive responses by regulating the inhibitory process^30,31^. Taking these data together, we hypothesized that altered mSFG synchronization in patients with diphasic dyskinesia during the ON state after LD medication may reflect the close relationship between deficits in inhibitory control of the mSFG and severity of dyskinetic symptoms. The mSFG, which is located in the prefrontal cortex (PFC), has been shown to be a key node in the cortico-striato-cortical circuit^32^ and dysfunction in this circuit has been reported to contribute to LID in PD patients^2,33^. Based on this evidence, we further speculated that abnormal synchronization in the mSFG might induce diphasic dyskinesia by damaging cortico-striato-cortical circuits.

However, the interactive analysis showed increased ReHo values in the left mSFG during the ON state in patients without dyskinesia when compared with those patients with diphasic dyskinesia. Our results show that these two types of PD patient have different changes in functional synchronization in the left mSFG during the response to LD. Apart from modulating the inhibition response, the SFG, as part of the PFC, is also critically involved in goal-directed processes^34^. The SFG is also a core node in the executive control network^35^, which activates or generates actions^36,37^. When LD is having an effect, the activated mSFG would thus facilitate improvements in motor symptoms in patients, without dyskinesia. Poorer performance of the mSFG in patients with diphasic dyskinesia, on the other hand, triggered the appearance of dyskinetic symptoms during the ON state. Both previous studies^38^ and our present study indicate that some PD patients can obtain motor benefits from LD for a long time without developing LID, showing that LD medication and dyskinesia do not always coexist. Taking these results together, we inferred that different changes in functional synchronization in the left mSFG triggered by the LD response in the early stage of PD might reflect the risk of diphasic dyskinesia in the later stages. This hypothesis needs to be confirmed in a longitudinal study.

Interestingly, moderation analyses showed that LEDD was not a significant moderator in the above interactive relationship. That is to say, the association between pathological inhibitory control of the mSFG and the severity of diphasic dyskinetic symptoms was independent of LD dose. In agreement with the present study, long-term LD treatment has been shown to result in maladaptive plastic alterations in the brain and, once LID had emerged, the involuntary movements gradually increased no matter how many doses of LD were used^39^. The severity of peak-dose dyskinesia, which is different from diphasic dyskinesia, was, however, correlated with LEDD and duration of LD use^1,40^.

Almost all previous studies focused on alterations in cerebral function in peak-dose dyskinesia. Cerasa et al.^4–6^ found that dysfunction in the inferior frontal gyrus is associated with peak-dose dyskinesia, whereas Herz et al.^16,41^ found that aberrant dopaminergic modulation between the putamen and primary motor cortex, or the pre-supplementary motor area, plays a key underlying role in peak-dose dyskinesia. Peak-dose dyskinesia and diphasic dyskinesia are both types of LID, which may suggest some similarities between the two. The notion that underactivity in prefrontal areas contributes to the development of dyskinetic symptoms is supported both by previous studies in peak-dose dyskinesia^4,5,42^ and by our study in diphasic dyskinesia. In terms of clinical manifestations, however, there are many differences between peak-dose dyskinesia and diphasic dyskinesia. For example, peak-dose dyskinesia occurs around the time of peak plasma levels of LD, whereas diphasic dyskinesia occurs at the beginning or end of the LD effect^1^. As another example, the appearance of peak-dose dyskinesia is usually choreoathetoid, but can also be ballistic or dystonic^1,40^, whereas diphasic dyskinesia often presents as repetitive alternating stereotypic movements^1^. It can, therefore, be inferred that the mechanism underlying diphasic dyskinesia is different from that underlying peak-dose dyskinesia^5^. Our study supports the idea that deficits in inhibitory control in the mSFG are related to the development of dyskinetic symptoms in patients with diphasic dyskinesia, whereas dysfunction in the inferior frontal gyrus is reported to be associated with peak-dose dyskinesia^5^. Although deficits in the right hemisphere have been reported to contribute to peak-dose dyskinesia^4,5^, we found that diphasic dyskinesia was associated with damage in the left hemisphere. The difference in alterations of brain function between patients with diphasic dyskinesia and those with peak-dose dyskinesia should be explored in further studies.

Some limitations should be taken into consideration when interpreting our results. First, only PD patients with diphasic dyskinesia were recruited in the study. To really understand the differences in pathophysiology between diphasic dyskinesia and peak-dose dyskinesia, a group of patients with peak-dose dyskinesia should be included in a future study. Second, we only explored neural functional alterations using the ReHo approach in rs-fMRI. Future studies should collect more comprehensive information using multimodal techniques, for example, functional connectivity or dynamic causal modeling in fMRI and cortical thickness or diffusion tensor imaging in structural MRI. Third, a comprehensive neuropsychological evaluation was not obtained in our study, reducing our ability to find specific cognitive impairment. We will recruit larger samples and use comprehensive neuropsychological testing to determine the underlying associations in cognitive status between PD patients with and without diphasic dyskinesia in the future work. Finally, the present cross-sectional study cannot show causal relationships, which need to be explored more thoroughly in a longitudinal study. In conclusion, the present study demonstrates that abnormal local synchronization in the left mSFG is a likely mechanism underlying diphasic dyskinesia and may provide new perspectives for further studies.

## Methods

### Participants

In total, 30 PD patients with diphasic dyskinesia and 23 PD patients without any type of LID, all of whom were treated with antiparkinsonism drugs including LD and were right-handed, participated in our study. Inclusion criteria were: (1) clinical diagnosis of PD according to UK Parkinson’s Disease Society Brain Bank criteria^43^; (2) positive longstanding response to LD and stable dose of antiparkinsonism drugs for at least 4 weeks prior to study; (3) presence or absence of diphasic dyskinesia (occurring at the beginning and end of the LD effect), observed by two experienced neurologists, following acute LD test or during chronic LD treatment, at last visit at least one week before MRI examination, together with previous relevant clinical symptoms; (4) no history of peak-dose dyskinesia (occurring at the peak of the LD effect) or off-period dystonia; (5) no current use of antidepressants, anxiolytics or antipsychotic drugs; (6) MMSE scores >24; (7) no evidence of other severe acute or chronic diseases, including stroke, brain tumor or psychiatric disease, and no contraindications for MRI scans. All patients had to be able to tolerate withdrawal of medication before scanning. We calculated the total LEDD for each PD patient^44^.

Each PD patient was fasted and received two resting-state functional magnetic resonance imaging (rs-fMRI) scans in the same morning. In order to determine the appropriate time for the second rs-fMRI scan, the patients were instructed to complete a diary card for one week before the scans. PD patients with diphasic dyskinesia who were taking antiparkinsonian drugs were asked to record on the diary card when the drugs began to work and stopped working, and when diphasic dyskinetic symptoms emerged and disappeared. PD patients without any type of LID who were taking antiparkinsonian drugs were asked to record on the diary card when the drugs worked and stopped working. The first rs-fMRI scan on each PD patient (functionally OFF) was performed at least 12 h after withdrawal of antiparkinsonian drugs. The patients then underwent clinical assessments, including MMSE, Hoehn and Yahr staging scale (H&Y) and motor component of the Unified Parkinson’s Disease Rating Scale (UPDRS-III), and afterwards took their antiparkinsonian drugs as usual. Patients with diphasic dyskinesia received a second fMRI scan (functionally ON) when dyskinetic symptoms were expected to occur, based on the previously completed diary cards. The fMRI scan was immediately stopped if abnormal involuntary movements would affect the scanning quality. However, no patient had severe dyskinetic symptoms that would influence the fMRI scan. Patients without dyskinesia received the second fMRI session when the antiparkinsonian drugs were working. Immediately after the ON-state fMRI scan, the severity of dyskinetic symptoms in patients with diphasic dyskinesia was evaluated using the Unified Dyskinesia Rating Scale (UDyRS).

All participants gave written informed consent before the start of the study, which was approved by the ethics committee of the First Affiliated Hospital of Nanjing Medical University.

### MRI acquisition

Participants were scanned using a 3.0 T Siemens MAGNETOM Verio whole-body MRI scanner (Siemens Medical Solutions, Germany), as described in our previous studies^45,46^. Tight foam padding was used to minimize head movement and ear-plugs were used to reduce noise. Participants were asked to remain as still as possible, close their eyes but remain awake, and try not to think about anything particular during the whole scanning procedure. Before acquiring functional scans, three-dimensional T1-weighted anatomical images were obtained using the following volumetric 3D magnetization-prepared rapid gradient-echo (MP-RAGE) sequence: (repetition time (TR) = 1900 ms, echo time (TE) = 2.95 ms, flip angle (FA) = 9°, slice thickness = 1 mm, slices = 160, field of view (FOV) = 230 × 230 mm², matrix size = 256 × 256). The first resting-state functional images on each participant were acquired using an echo-planar imaging sequence with the following parameters: TR = 2000 ms, TE = 21 ms, FA = 90°, FOV = 256 × 56 mm², in-plane matrix = 64 × 64, slices = 35, slice thickness = 3 mm, no slice gap, total volumes = 240. After taking antiparkinsonian drugs as mentioned above, only resting-state functional images were acquired during the second fMRI scan.

### Preprocessing of fMRI data

Preprocessing of fMRI data was similar to our previous study^46^. The images were preprocessed and analyzed using DPARSF software (http://www.restfmri.net/forum/dparsf)^47^. The first 10 time points were disregarded and the remaining 230 volumes were used for slice timing correction and head motion correction. Participants with head motions exceeding 2 mm, or 2° of translation, or rotation in any direction would be excluded, but no participant was excluded from the current study. We calculated mean head translation, mean head rotation and frame-wise displacement^48^. Analysis of these head motion parameters did not reveal any difference between the two groups during OFF or ON state (*P* > 0.05). High-resolution T1 structural images were coregistrated to functional images using a nonlinear image registration approach and segmented using a new segment algorithm with diffeomorphic anatomical registration through exponentiated lie algebra (DARTEL), followed by a 24 parameter Volterra expansion. Finally, fMRI images were spatially normalized into the Montreal Neurological Institute (MNI) template and resampled into a spatial resolution of 3 × 3 × 3 mm³. A temporal filter (0.01 Hz < *f* < 0.08 Hz) was used to remove low-frequency drifts and physiological high-frequency noise, with a finite impulse response filter.

### ReHo processing

Data without spatial smoothing were subjected to ReHo analysis using DPARSF software. In brief, as introduced previously^49^, ReHo maps were produced on a voxel-by-voxel basis by calculating Kendall’s coefficient of concordance (KCC) to compare the similarity of the time series of a given voxel with those of its nearest neighbors (26 voxels). To reduce the influence of individual variations in KCC value, the ReHo maps were normalized by dividing the KCC for each voxel by the average KCC for the whole brain. Finally, the data were smoothed with a Gaussian filter of 6 mm full width at half-maximum to suppress noise and effects due to residual differences in functional and gyral anatomy during inter-subject averaging.

### Statistical analysis

Demographic and clinical data were analyzed using IBM SPSS statistics v 20.0.0 software (SPSS, Chicago, IL, USA). Comparisons were made between diphasic dyskinetic and non-dyskinetic patients. A Kolmogorov–Smirnov test was used to test for normality in continuous variables and a two-sample *t* test was used for normally distributed data. Asymmetrically distributed variables were tested using the Mann–Whitney *U* test, as appropriate. A chi-square test was used for gender. A significance threshold was set at two-tailed *p* < 0.05.

Two-way factorial repeated measures analysis of covariance was performed with group (PD patients with diphasic dyskinesia or PD patients without dyskinesia) as a between-subject factor and LD effect (before or after taken antiparkinsonian drugs) as a within-subject factor, after adjusting for age, gender and education years, to explore two main effects and interaction. Specifically, the main group effect was determined by comparing ReHo values in diphasic dyskinetic patients with those in non-dyskinetic patients. Similarly, the main LD effect was assessed using ReHo values before or after taking antiparkinsonian drugs, regardless of disease status. Most importantly, the interactive analysis combined disease status and LD effect. All statistical thresholds were set at a corrected *p* < 0.01, determined by Monte Carlo simulation for multiple comparisons (http://afni.nimh.nih.gov/pub/dist/doc/manual/AlphaSim.pdf). Afterwards, post hoc analyses were performed and corrected by the Bonferroni correction with a significant different *p* < 0.013 (0.05/4 [number of pair-comparisons]). In addition, Spearman’s correlative analyses, with significance thresholds set at two-tailed *p* < 0.05, were performed between UDyRS scores and ReHo values of the clusters showing significant interaction between group and LD effect.

It has been proposed that the emergence of LID is associated with numerous factors, including non-modifiable risk factors, such as age, gender and disease duration, along with modifiable risk factors, such as LEDD^40^. As LEDD is the most important modifiable risk factor associated with the occurrence of LID^50,51^, in this study, we recruited closely matched diphasic dyskinetic and non-dyskinetic groups to minimize the possible influence of these clinical variables. To further explore the potential moderating effect of LEDD on associations between severity of dyskinetic symptoms and local synchronization changes in the above clusters, moderation analyses were performed using the SPSS macro PROCESS^52^. Within PROCESS, model 1 was selected and the confidence interval was set to 95%. For one cluster, two moderation models were built. In one moderation model, ReHo values were entered as the predictor (*X*), UDyRS scores as the outcome (*Y*), and LEDD was added as the moderator (M). In the second moderation model, LEDD was also added as the moderator (M), but UDyRS scores were entered as the predictor (X) and ReHo values as the outcome (*Y*). If a significant interaction between the predictor (*X*) and the moderator (*M*) emerged, the Johnson-Neyman Technique was then used to identify a significant influence of the moderator (*M*) on the association between the predictor (*X*) and the outcome (*Y*)^52^. All significance thresholds were set at two-tailed *p* < 0.05.

### Reporting summary

Further information on research design is available in the Nature Research Reporting Summary linked to this article.

## Supplementary information

Reporting Summary

## Data Availability

The data that support the findings of this study are available from the corresponding author upon reasonable request.

## Code availability

As previously mentioned in the MRI subsection, ReHo analysis was performed on Data Processing Assistant for Resting-State fMRI (DPARSF) and REST (http://restfmri.net).

## References

[1] Espay, A. J. et al. Levodopa-induced dyskinesia in Parkinson disease: current and evolving concepts. *Ann. Neurol.* **84**, 797–811 (2018). 10.1002/ana.25364 30357892

[2] Picconi B, Hernandez LF, Obeso JA, Calabresi P (2018). Motor complications in Parkinson’s disease: Striatal molecular and electrophysiological mechanisms of dyskinesias. Mov. Disord..

[3] Rothwell JC, Obeso JA (2015). Can levodopa-induced dyskinesias go beyond the motor circuit?. Brain.

[4] Cerasa A (2012). Prefrontal alterations in Parkinson’s disease with levodopa-induced dyskinesia during fMRI motor task. Mov. Disord..

[5] Cerasa A, Koch G, Donzuso G, Mangone G (2015). A network centred on the inferior frontal cortex is critically involved in levodopa-induced dyskinesias. Brain.

[6] Cerasa A (2015). The motor inhibition system in Parkinson’s disease with levodopa-induced dyskinesias. Mov. Disord..

[7] Herz DM (2016). Resting-state connectivity predicts levodopa-induced dyskinesias in Parkinson’s disease. Mov. Disord..

[8] Zhong Y (2011). Altered regional synchronization in epileptic patients with generalized tonic-clonic seizures. Epilepsy Res..

[9] Jiang L (2015). Toward neurobiological characterization of functional homogeneity in the human cortex: regional variation, morphological association and functional covariance network organization. Brain Struct. Funct..

[10] Homayoun H (2018). Parkinson Disease. Ann. Intern. Med..

[11] Tziortzi AC (2014). Connectivity-based functional analysis of dopamine release in the striatum using diffusion-weighted MRI and positron emission tomography. Cereb. Cortex.

[12] Kreitzer AC, Malenka RC (2008). Striatal plasticity and basal ganglia circuit function. Neuron.

[13] Surmeier DJ, Ding J, Day M, Wang Z, Shen W (2007). D1 and D2 dopamine-receptor modulation of striatal glutamatergic signaling in striatal medium spiny neurons. Trends Neurosci..

[14] Mazzoni P, Hristova A, Krakauer JW (2007). Why don’t we move faster? Parkinson’s disease, movement vigor, and implicit motivation. J. Neurosci..

[15] Kish SJ, Shannak K, Hornykiewicz O (1988). Uneven pattern of dopamine loss in the striatum of patients with idiopathic Parkinson’s disease. Pathophysiologic and clinical implications. N. Engl. J. Med..

[16] Herz DM (2014). The acute brain response to levodopa heralds dyskinesias in Parkinson disease. Ann. Neurol..

[17] Lorenz RC (2015). Interactions between glutamate, dopamine, and the neuronal signature of response inhibition in the human striatum. Hum. Brain Mapp..

[18] Harrington DL (2018). Altered functional interactions of inhibition regions in cognitively normal Parkinson’s disease. Front. Aging Neurosci..

[19] Turner RS, Grafton ST, McIntosh AR, DeLong MR, Hoffman JM (2003). The functional anatomy of parkinsonian bradykinesia. Neuroimage Clin..

[20] Gilat M (2017). Dopamine depletion impairs gait automaticity by altering cortico-striatal and cerebellar processing in Parkinson’s disease. Neuroimage.

[21] Esposito F (2013). Rhythm-specific modulation of the sensorimotor network in drug-naive patients with Parkinson’s disease by levodopa. Brain.

[22] Berman BD (2016). Levodopa modulates small-world architecture of functional brain networks in Parkinson’s disease. Mov. Disord..

[23] Goetz CG, Nutt JG, Stebbins GT (2008). The Unified Dyskinesia Rating Scale: presentation and clinimetric profile. Mov. Disord..

[24] Goetz CG (2011). Temporal stability of the Unified Dyskinesia Rating Scale. Mov. Disord..

[25] Dambacher F (2014). The role of right prefrontal and medial cortex in response inhibition: interfering with action restraint and action cancellation using transcranial magnetic brain stimulation. J. Cogn. Neurosci..

[26] Chmielewski WX, Muckschel M, Dippel G, Beste C (2016). Concurrent information affects response inhibition processes via the modulation of theta oscillations in cognitive control networks. Brain Struct. Funct..

[27] Picton TW (2007). Effects of focal frontal lesions on response inhibition. Cereb. Cortex.

[28] Bari A, Robbins TW (2013). Inhibition and impulsivity: behavioral and neural basis of response control. Prog. Neurobiol..

[29] Picazio S, Ponzo V, Caltagirone C, Brusa L, Koch G (2018). Dysfunctional inhibitory control in Parkinson’s disease patients with levodopa-induced dyskinesias. J. Neurol..

[30] Zois E (2017). Frontal cortex gray matter volume alterations in pathological gambling occur independently from substance use disorder. Addict. Biol..

[31] Hu S, Ide JS, Zhang S, Li CR (2016). The right superior frontal gyrus and individual variation in proactive control of impulsive response. J. Neurosci..

[32] Otis JM (2017). Prefrontal cortex output circuits guide reward seeking through divergent cue encoding. Nature.

[33] Barroso-Chinea, P. & Bezard, E. Basal Ganglia circuits underlying the pathophysiology of levodopa-induced dyskinesia. *Front. Neuroanat.* **4**, 131 (2010). 10.3389/fnana.2010.00131 PMC2947938 20890450

[34] Egner T, Hirsch J (2005). The neural correlates and functional integration of cognitive control in a Stroop task. Neuroimage.

[35] Li W (2013). Subregions of the human superior frontal gyrus and their connections. Neuroimage.

[36] Duncan J (2013). The structure of cognition: attentional episodes in mind and brain. Neuron.

[37] Duncan J (2010). The multiple-demand (MD) system of the primate brain: mental programs for intelligent behaviour. Trends Cogn. Sci..

[38] Nutt JG, Chung KA, Holford NHG (2010). Dyskinesia and the antiparkinsonian response always temporally coincide: A retrospective study. Neurology.

[39] Brotchie JM (2005). Nondopaminergic mechanisms in levodopa-induced dyskinesia. Mov. Disord..

[40] Tran TN, Vo TNN, Frei K, Truong DD (2018). Levodopa-induced dyskinesia: clinical features, incidence, and risk factors. J. Neural Transm. (Vienna).

[41] Herz DM (2015). Abnormal dopaminergic modulation of striato-cortical networks underlies levodopa-induced dyskinesias in humans. Brain.

[42] Cerasa A (2015). A network centred on the inferior frontal cortex is critically involved in levodopa-induced dyskinesias. Brain.

[43] Hughes AJ, Daniel SE, Kilford L, Lees AJ (1992). Accuracy of clinical diagnosis of idiopathic Parkinson’s disease: a clinico-pathological study of 100 cases. J. Neurol. Neurosurg. Psychiatry.

[44] Tomlinson CL (2010). Systematic review of levodopa dose equivalency reporting in Parkinson’s disease. Mov. Disord..

[45] Shen YT (2018). Disrupted amplitude of low-frequency fluctuations and causal connectivity in Parkinson’s disease with apathy. Neurosci. Lett..

[46] Shen YT (2019). BST1 rs4698412 allelic variant increases the risk of gait or balance deficits in patients with Parkinson’s disease. CNS Neurosci. Ther..

[47] Chao-Gan Y, Yu-Feng Z (2010). DPARSF: a MATLAB toolbox for “Pipeline” data analysis of resting-state fMRI. Front Syst. Neurosci..

[48] Power JD, Barnes KA, Snyder AZ, Schlaggar BL, Petersen SE (2012). Spurious but systematic correlations in functional connectivity MRI networks arise from subject motion. Neuroimage.

[49] Zang Y, Jiang T, Lu Y, He Y, Tian L (2004). Regional homogeneity approach to fMRI data analysis. Neuroimage.

[50] Warren Olanow C (2013). Factors predictive of the development of Levodopa-induced dyskinesia and wearing-off in Parkinson’s disease. Mov. Disord..

[51] Hauser RA, McDermott MP, Messing S (2006). Factors associated with the development of motor fluctuations and dyskinesias in Parkinson disease. Arch. Neurol..

[52] Hayes AF (2013). Introduction to mediation, moderation, and conditional process analysis: a regression-based approach. J. Educ. Meas..

