# Supplementary material for: Dysfunction in superior frontal gyrus associated with diphasic dyskinesia in Parkinson’s disease
Source: NPJ Parkinsons Dis. 2020 Oct 30;6:30. doi: 10.1038/s41531-020-00133-y (PMC7603392; doi:10.1038/s41531-020-00133-y)
Supplement: Supplementary file 1 — Reporting Summary [file 41531_2020_133_MOESM1_ESM.pdf]

nature research

Corresponding author(s): NPJPARKD-00426R

Last updated by author(s): Jul 11, 2020

Reporting Summary

Nature Research wishes to improve the reproducibility of the work that we publish. This form provides structure for consistency and transparency in reporting. For further information on Nature Research policies, see Authors & Referees and the Editorial Policy Checklist.

Statistics

For all statistical analyses, confirm that the following items are present in the figure legend, table legend, main text, or Methods section.

n/a	Confirmed	
☐	☒	The exact sample size (*n*) for each experimental group/condition, given as a discrete number and unit of measurement
☐	☒	A statement on whether measurements were taken from distinct samples or whether the same sample was measured repeatedly
☐	☒	The statistical test(s) used AND whether they are one- or two-sided<br>*Only common tests should be described solely by name; describe more complex techniques in the Methods section.*
☐	☒	A description of all covariates tested
☐	☒	A description of any assumptions or corrections, such as tests of normality and adjustment for multiple comparisons
☐	☒	A full description of the statistical parameters including central tendency (e.g. means) or other basic estimates (e.g. regression coefficient) AND variation (e.g. standard deviation) or associated estimates of uncertainty (e.g. confidence intervals)
☐	☒	For null hypothesis testing, the test statistic (e.g. *F*, *t*, *r*) with confidence intervals, effect sizes, degrees of freedom and *P* value noted<br>*Give P values as exact values whenever suitable.*
☒	☐	For Bayesian analysis, information on the choice of priors and Markov chain Monte Carlo settings
☐	☒	For hierarchical and complex designs, identification of the appropriate level for tests and full reporting of outcomes
☐	☒	Estimates of effect sizes (e.g. Cohen's *d*, Pearson's *r*), indicating how they were calculated

Our web collection on statistics for biologists contains articles on many of the points above.

Software and code

Policy information about availability of computer code

Data collection	no software was used
Data analysis	DPARSF and REST

For manuscripts utilizing custom algorithms or software that are central to the research but not yet described in published literature, software must be made available to editors/reviewers. We strongly encourage code deposition in a community repository (e.g. GitHub). See the Nature Research guidelines for submitting code & software for further information.

Data

Policy information about availability of data

All manuscripts must include a data availability statement. This statement should provide the following information, where applicable:

- Accession codes, unique identifiers, or web links for publicly available datasets
- A list of figures that have associated raw data
- A description of any restrictions on data availability

The data that support the findings of this study are available from the corresponding author upon reasonable request.

Field-specific reporting

Please select the one below that is the best fit for your research. If you are not sure, read the appropriate sections before making your selection.

☒ Life sciences ☐ Behavioural & social sciences ☐ Ecological, evolutionary & environmental sciences

For a reference copy of the document with all sections, see nature.com/documents/nr-reporting-summary-flat.pdf

Life sciences study design

All studies must disclose on these points even when the disclosure is negative.

Sample size	We enrolled 30 PD patients with and 23 PD patients without any type of LID. The incidence of Parkinson's disease is about 1.7%, and LIDs occurred in 40% PD patients after 4-6 years of levodopa treatment. We think our sample size are sufficient.
Data exclusions	No participant was excluded from the current study.
Replication	All attempts at replication were successful.
Randomization	We collected all participants information, including age, gender and education years. And further used them as covariates during the analyses.
Blinding	Our study aimed to study the neural mechanisms of diphasic dyskinesia in Parkinson's disease. And the investigators were grouped according to their own disease status.

Reporting for specific materials, systems and methods

We require information from authors about some types of materials, experimental systems and methods used in many studies. Here, indicate whether each material, system or method listed is relevant to your study. If you are not sure if a list item applies to your research, read the appropriate section before selecting a response.

Materials & experimental systems

n/a	Involved in the study	
☒	☐	Antibodies
☒	☐	Eukaryotic cell lines
☒	☐	Palaeontology
☒	☐	Animals and other organisms
☐	☒	Human research participants
☒	☐	Clinical data

Methods

n/a	Involved in the study	
☒	☐	ChIP-seq
☒	☐	Flow cytometry
☐	☒	MRI-based neuroimaging

Human research participants

Policy information about studies involving human research participants

Population characteristics	For PD patients with diphasic dyskinesia, 13 were females and 17 were males. Their average was 63.83. For PD patients without dyskinesia, 8 were females and 15 were males. Their average was 61.52. All of them were treated with antiparkinsonism drugs including LD and were right-handed.
Recruitment	Patients were recruited from outpatient patients who went to the hospital for treatment.
Ethics oversight	This study was approved by the ethics committee of the First Affiliated Hospital of Nanjing Medical University.

Note that full information on the approval of the study protocol must also be provided in the manuscript.

Magnetic resonance imaging

Experimental design

Design type	resting-state
Design specifications	Each PD patient was fasted and received two resting-state functional magnetic resonance imaging (rs-fMRI) scans in the same morning. The first scan (practical "OFF" phase) of our experiment was acquired after 12h withdrawal of all dopaminergic medication. Then patients took their usual morning levodopa dose and the second MRI scan ("ON" phase) was performed when the patients responded to levodopa as expected.
Behavioral performance measures	The patients underwent clinical assessments, including MMSE, Hoehn and Yahr staging scale (H&Y) and motor component of the Unified Parkinson's Disease Rating Scale (UPDRS-III).

Acquisition

Imaging type(s)	functional
Field strength	3.0 T
Sequence & imaging parameters	Three-dimensional T1-weighted anatomical images were acquired using the following volumetric 3D magnetization-prepared rapid gradient-echo (MP-RAGE) sequence with the following parameters: repetition time [TR] = 1900 ms, echo time [TE] = 2.95 ms, flip angle [FA] =9°, slice thickness = 1 mm, slices = 160, field of view [FOV] = 230 × 230 mm2, matrix size = 256 × 256 and voxel size = 1 × 1 × 1 mm3. Resting-state functional images were collected using an echo-planar imaging (EPI) sequence with the following parameters: TR = 2000 ms, TE = 21 ms, FA = 90°, FOV = 256 × 256 mm2, in-plane matrix = 64 × 64, slices = 35, slice thickness = 3 mm, no slice gap, voxel size = 3 × 3 × 3 mm3, total volumes = 240.
Area of acquisition	whole brain scan

Diffusion MRI ☐ Used ☒ Not used

Preprocessing

Preprocessing software	MATLAB software (version R2016b, Math Works, Inc., Natick, MA, USA)<br>DPARSF, http://www.restfmri.net/forum/dparsf<br>REST, http://restfmri.net
Normalization	DARTEL normalization was applied to compute the transformations from the native space to the Montreal Neurological Institute (MNI) space.
Normalization template	MNI space
Noise and artifact removal	Several sources of spurious variance were regressed out, including the white matter signal, the cerebral spinal fluid signal, and six head motion parameters obtained by head-motion correction.
Volume censoring	SPM12 toolbox (http://www.fil.ion.ucl.ac.uk/spm/)

Statistical modeling & inference

Model type and settings	Case controlled study
Effect(s) tested	have used ANOVA analysis

Specify type of analysis: ☒ Whole brain ☐ ROI-based ☐ Both

Statistic type for inference<br>(See Eklund et al. 2016)	voxel-level p < 0.01, a cluster-level significance threshold of p < 0.01 determined by a Monte Carlo simulation
Correction	Monte Carlo

Models & analysis

n/a	Involved in the study
☐	☒ Functional and/or effective connectivity
☒	☐ Graph analysis
☒	☐ Multivariate modeling or predictive analysis

Functional and/or effective connectivity	Spearman’s correlative analyses
